# Effects of Irrigation at Different Fractions of Crop Evapotranspiration on Water Productivity and Flavonoid Composition of Cabernet Sauvignon Grapevine

**DOI:** 10.3389/fpls.2021.712622

**Published:** 2021-09-01

**Authors:** Nazareth Torres, Runze Yu, Johann Martínez-Lüscher, Evmorfia Kostaki, Sahap Kaan Kurtural

**Affiliations:** ^1^Department of Viticulture and Enology, University of California, Davis, Davis, CA, United States; ^2^Department of Agronomy, Biotechnology and Food Science, Public University of Navarra, Pamplona, Spain; ^3^Semios Biotechnologies Toronto, Toronto, ON, Canada

**Keywords:** arbuscular mycorrhizal fungi, berry quality, climate change, deficit irrigation, water footprint, water scarcity, water use efficiency

## Abstract

Climate change models predict lower precipitation and higher air temperatures that will negatively affect viticultural regions. Irrigation of vineyards will be crucial for mitigating abiotic stress during the growing season. However, the environmental impact of irrigation requires consideration for ensuring its sustainability in the future. We evaluated the standard irrigation practices on grapevine water use efficiency, berry flavonoid composition, vineyard water footprint, and arbuscular mycorrhizal fungi-grapevine symbiosis in two seasons with contrasting amounts of precipitation. The irrigation treatments consisted of weekly replacement of 25, 50, and 100% of crop evapotranspiration (ET_c_) during two growing seasons. Irrigation in grapevine vineyards mitigated the water scarcity when precipitation during the dormant season was not sufficient. The results provided field data supporting that despite the low rainfall recorded in one of the seasons, increasing the amount of irrigation was not advised, and replacing 50% ET_c_ was sufficient. In this treatment, berry composition was improved with increased contents of total soluble solids, anthocyanins, and flavonols, and a stable flavonoid profile without an economic decrease in yield. In addition, with 50% ET_c_, the mycorrhizal symbiosis was not compromised and water resources were not highly impacted. Altogether, our results provide fundamental knowledge for viticulturists to design an appropriate irrigation schedule under the future warming scenarios with minimal environmental impact in semi-arid regions facing warming trends.

## Introduction

Global warming trends due to climate change are likely to continue at the current rate, leading to temperature increases of 1.5–4.5°C between 2030 and 2052 (IPCC et al., 2018). Furthermore, changes in precipitation patterns, frequencies of heatwaves, droughts, and a general increase in evapotranspiration (ET) rates are also expected (IPCC et al., 2018). These changes would in turn, affect soil moisture, ground water table, storage of water in reservoirs, and the salinization of shallow aquifers (Bates et al., 2008).

The effects of climate change are leading to an increasingly negative water balance during the grapevine growing season (Martínez-Lüscher et al., 2017, 2020). The effects have been an advance of grapevine phenology, diminution of yield and a lack of cultivar trait expression at the farm gate (Costa et al., 2016; Mosedale et al., 2016; Torres et al., 2018a; Venios et al., 2020). Different approaches have been used in recent years to cope with these effects, such as the use of drought-tolerant rootstocks, clones and/or varieties, improved training systems or increased row spaces, and irrigation applications (van Leeuwen et al., 2019). Although winegrowers still prioritize canopy and soil management and changes in harvest date and winemaking techniques over water management (Neethling et al., 2017), use of irrigation in vineyards is inevitable in historically non-irrigated areas due to the warming trends (Costa et al., 2016; Resco et al., 2016). Furthermore, many of the viticulture areas of the world rely on irrigation for consistent production (Torres et al., 2021b), such as California, where irrigation is required to overcome the dry and warm summers. Thus, irrigated agriculture in California is the largest consumer of fresh water, which accounted for about 50% of the total water supplied to the state in 2011–2015 (CDWR, 2019). However, water resources, particularly groundwater, have reached a critical state due to extended drought periods, and overuse by irrigated agriculture (Wilson et al., 2020). In fact, Alam et al. (2019) recently reported that increases in water demand and decreases in surface water supply caused by a warming climate might negatively affect groundwater storage, especially in regions like the San Joaquin Valley of California, where groundwater reserve is already stressed. Therefore, evidence support the necessity of finding sustainable practices in vineyard production systems for long-time exploitation of natural resources, such as water and soil.

Water footprint (WF) is the volume of water used per unit of food produced. It was suggested as an indicator of the total water used for grape production to promote sustainable and efficient use of water in viticulture (Mekonnen and Hoekstra, 2011). WF is the sum of three components; the green WF or water from precipitation, the blue WF or irrigation water sourced from surface or groundwater resources, and the gray WF that is the amount of fresh water required to assimilate pollutants to meet specific water quality standards (Mekonnen and Hoekstra, 2011). The agriculture sector accounted for 92% of the total WF (about 8,360 billion m^3^/year; Mekonnen and Hoekstra, 2011). The WF of irrigated crops recently came under scrutiny due to socioeconomic concerns and the need to reduce it (Cominelli et al., 2009; D'Ambrosio et al., 2020). Intrinsic water use efficiency (_i_WUE) is the ratio of moles of CO_2_ assimilated and moles of water transpired by the plant (Tomás et al., 2014). It is an indicator of how efficient the grapevine is, utilizing water to produce photosynthates. Deficit irrigation strategies were developed to reduce the amount of water applied to the grapevine, substantially reducing the WF and increasing the _i_WUE. The other aim in applying water deficits was to maintain or improve grape berry composition (Terry and Kurtural, 2011). Deficit irrigation methods apply a predetermined fraction of the crop evapotranspiration (ET_c_), during a portion of the growing season (Torres et al., 2021a). A large and growing body of literature investigated how these strategies, including various timing, duration, and severity, affected grapevine physiology and consequently, berry composition regarding sugar and anthocyanin accumulation and the subsequent chemical composition in wines (Chaves et al., 2010, Intrigliolo and Castel, 2008; Keller et al., 2016; da Silva et al., 2018; Torres et al., 2021b). Nevertheless, the WF of these strategies at a local scale in warm climates has not been addressed thoroughly.

Arbuscular mycorrhizal fungi (AMF) are soil-borne fungi that establish mutualistic relationships with terrestrial plants including grapevines, being key components of the viticulture production systems (Trouvelot et al., 2015; Torres et al., 2018a). The symbiosis of grapevines with AMF may be affected by stress factors related to climate change (Compant et al., 2010). In addition, previous studies demonstrated that management practices strongly shaped bacterial and fungal communities in vineyard soils (Coller et al., 2019; Vink et al., 2021), which may modulate grapevine responses to environmental stresses. Under controlled conditions, the association of grapevines with AMF enhanced berry quality (i.e., increased phenolic content) when potted grapevines were subjected to deficit irrigation and elevated temperature (Torres et al., 2018b,c), whereas berry flavonoid metabolism was upregulated in AMF-inoculated grapevines in vineyards (Torres et al., 2021a).

Previous work indicated that different replacements of the ET_c_ affected grapevine physiology (water status and gas exchange parameters), leading to a different carbon allocation between source and sink organs (Torres et al., 2021b). This study also demonstrated that replacing 50% of the ET_c_ was sufficient to sustain the grapevine performance through the enhancement of sugar transport that could slow down the detrimental effect of water deficits on yield (Torres et al., 2021b). Based on the above-mentioned literature, it was hypothesized that (1) water deficits may increase grapevine iWUE, promoting the balance between vegetative and reproductive growth and improving berry composition in a hot climate and (2) deficit irrigation strategies may exert a different pressure on water resources and AMF associated with grapevines. Therefore, the aim of this study was to evaluate three applied water amounts based on different fractions of the ET_c_ for maintaining berry quality without compromising yield and minimizing their environmental impact concerning total WF and AMF colonization rates. This work covered the effect of irrigation strategies on different interrelated elements of vineyard production systems such as the soil water storage or the AMF abundance together with the productive characteristics in terms of yield and quality, advancing in the knowledge of a more sustainable water management.

## Materials and Methods

### Plant Material and Experimental Design

The experiment was conducted on Cabernet Sauvignon (clone FPS08) on 110R rootstock during two consecutive seasons (2018–2019 to 2019–2020) in Oakville, CA (38.428°N, 122.409°W). Grapevines were planted in 2011 with a spacing of 2.4 by 2.0 m (row × vine) with a row orientation of North West-South East. The grapevines were trained to a bilateral cordon on a vertically shoot positioned trellis with a cordon height 96 cm above vineyard floor and pruned to 30 spurs (15 spurs/m) and one bud per spur. The experiment was designed as a randomized complete block with a one-way arrangement of the following fractions of ET_c_ replacement treatments: (i) 25% ET_c_, (ii) 50% ET_c_, and (iii) 100% ET_c_. Each treatment was replicated six times with five grapevines in each treatment replicate. The three middle vines within the treatment replicate were used for data collection and the two on distal ends were treated as buffer plants. Plants were irrigated weekly with two drip emitters per vine. Other cultural practices were standard for the area and conducted before treatment application.

### Weather Data and Applied Water Amounts Treatments

**Figure 1:**
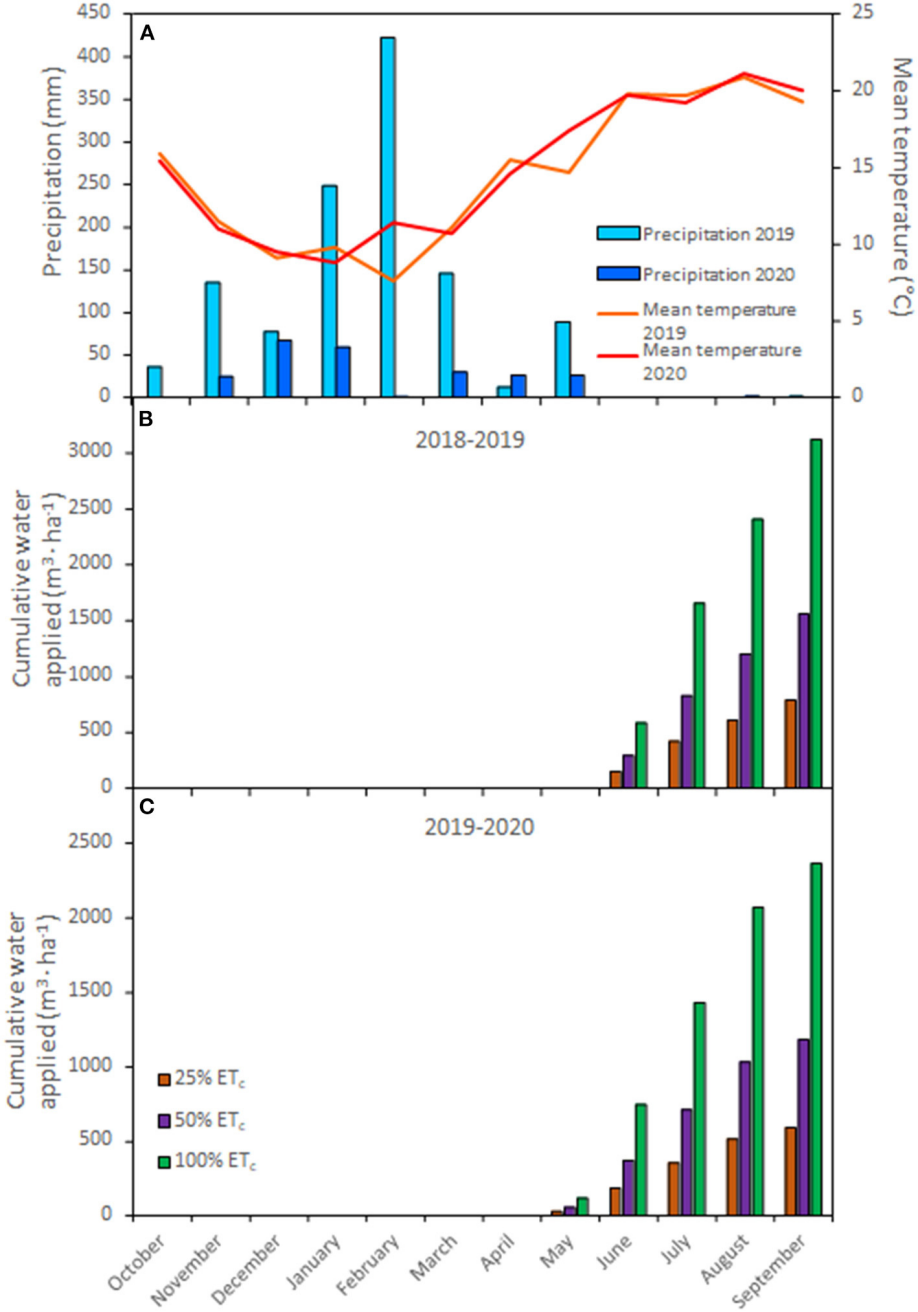
Mean air temperature (°C) and precipitation (mm) during the 2018–2019 and 2019–2020 growing seasons **(A)** Weather data were obtained from the CIMIS weather station # 77 (Oakville, CA) located at the research site. Cumulative water applied (m^3^ · ha^−1^) to Cabernet Sauvignon grapevines (clone FPS08) according to the different replacements of crop evapotranspiration (25, 50, and 100% ET_c_) during the 2018–2019 **(B)** and 2019–2020 **(C)** growing seasons in Oakville, CA.

Weather data (Figure 1A) were obtained from the California Irrigation Management Information System (CIMIS, station #77, Oakville, CA) located 160 m from the experimental vineyard (CIMIS, 2020).

The applied water amounts treatments consisted of replacing different fractions of ET_c_. Vineyard ET_c_ was calculated using the following equation: ET_c_ = ET_o_ × K_c_, where ET_o_ is the reference ET and K_c_ is the crop coefficient. The ET_o_ was measured by the California Irrigation Monitoring Information System station 77, 160 m from this experimental vineyard. The evaluation of vineyard K_c_ over the course of the growing season was calculated using the shade cast beneath grapevines grown with 100% ET_o_ application from April 1 and the vertical shoot-positioned (VSP)-specific equation developed by Williams (2014) and adjusted for row spacing. The irrigation treatments were imposed when stem water potential was lower than −1 MPa combined with a visual assessment of the first symptoms of water stress in petioles and leaves (June 2019 and May 2020) to harvest (no irrigation was used after harvest) of each growing season (Figures 1B,C). Different irrigation treatments were implemented through the variation of the emitter output per grapevine in the drip line from 8 L · h^−1^ to 2 L · h^−1^ and 4 L · h^−1^, respectively, and the irrigation pump was scheduled to run based on the application rate of the 100% ET_c_ treatment plots. Thus, the 25, 50, and the 100% ET_c_ plots received 4 L · h^−1^, 8 L · h^−1^, and 16 L · h^−1^ per grapevine, respectively.

### _i_WUE and Crop Water Use Efficiency

Leaf gas exchange was measured with a CIRAS-3 portable infrared gas analyzer system (PP Systems, Amesbury, MA, USA) featuring a broad-leaf chamber with a 4.5 cm^2^ window. For each sample date and treatment replicate, three measurements were made *ca*. solar noon (11:30 to 13:30 h) on a healthy and fully expanded leaf within a minute to obtain an accurate range of leaf gas exchange and then, values were averaged. The cuvette was oriented perpendicularly to sunlight, which was always in saturating conditions (average of internal PAR = 1969 ± 135 μmol · m^−2^ s^−1^). Chamber conditions were set up at 40% relative humidity, a CO_2_ concentration of 400 μmol·mol^−1^, and using a flow to the chamber of 300 mL·min^−1^. The _i_WUE was calculated as the ratio between carbon assimilation rate (A_N_) and stomatal conductance (g_s_) and expressed as μmol CO_2_ ·mmol H_2_O^−1^. The crop water use efficiency (WUE_c_) was calculated as the ratio between yield expressed as kg·ha^−1^ and the amount of water applied to each plot (m^3^ · ha^−1^) as reported by Medrano et al. (2015).

### Carbon Isotope Composition of Musts (δ^13^C)

Carbon stable isotope composition (δ^13^C) was measured in the berry must at harvest following the protocol described by Gaudillère et al. (2002). A 2 mL aliquot was centrifuged at 14,119 × *g* to remove suspended solids. Then, 5 μL of the supernatant was pipetted in thin capsules, dried overnight at 60°C, and encapsulated using tweezers. Isotopic analyses were performed at the UC Davis Stable Isotope facility, using a PDZ Europa ANCA-GSL elemental analyzer interfaced to a PDZ Europa 20–20 isotope ratio mass spectrometer (Sercon Ltd., Cheshire, United Kingdom) following the methods described in Brillante et al. (2020). Results for each of the samples were normalized to the reference standard MAB using the following equation (1) and expressed in delta notation:

$$\begin{array}{l} Eq.1.\;\delta^{13}C=\left[\frac{Rsample}{Rstandard}-1\right]\times \;1000 \end{array}$$

where R_sample_ and R_standard_ are the absolute ^13^C/^12^C ratios for sample and standard. The values of δ^13^C are reported in parts per thousand with respect to the Vienna Pee Dee Belemnite (VPDB) international reference.

### Yield Components and Leaf Biomass

The harvest commenced when the berry total soluble solids (TSS) reached *ca*. 24°Brix on an average in all treatments on September 25, 2019 [114 days after flowering (DAF)] and September 8, 2020 (115 DAF), respectively. Clusters per vine were counted and weighed on a top-loading balance. One vine per treatment replicate was then defoliated and leaves were weighed to obtain leaf mass per area unit (tonnes · ha^−1^). Leaf area was measured with a LI-3100 Area meter (LI-COR, Lincoln, NE, USA) on a subsample of leaves and then it was related to dry mass (e.g., *via* specific leaf area (SLA) cm^2^ · g^−1^). The total dry mass of leaves collected within a known ground surface area was converted into leaf area index (LAI) by multiplying with the SLA. In the following winter of each season, shoots were pruned, and weighed on a top-loading balance to determine pruning mass. The yield to pruning mass ratio was calculated as the ratio between the yield and the pruning mass in the following dormant season.

### Berry Size and Primary Metabolites

At harvest, 60 berries were randomly collected from the three middle grapevine of each replicate (*n* = 6) during both growing seasons and immediately processed. Berries were weighed, averaged, and berry mass was obtained during the growing season. At harvest, berries were gently pressed by hand to express the juice. The TSS were determined using a temperature-compensating digital refractometer (Atago PR-32, Bellevue, WA, USA). Must pH and titratable acidity (TA) were determined with an autotitrator (Metrohm 862 Compact Titrosampler, Herisau, Switzerland). TA was estimated by titration with 0.1 N sodium hydroxide to an end point of 8.3 pH and reported as g · L^−1^ of tartaric acid.

### Berry Skin Flavonoid Composition

Berry skin flavonoid composition was determined in 20 berries randomly collected from each treatment replicate (*n* = 6). Berries were gently peeled and skins were freeze-dried (Cold Trap 7,385,020, Labconco, Kansas City, MO, USA) and ground with a tissue lyser (MM400, Retsch, Germany). Fifty (50) mg of the resultant powder was extracted in methanol: water: 7 M hydrochloric acid (70:29:1, V:V:V) to simultaneously determine flavonol and anthocyanin concentration and profile as previously described in Martínez-Lüscher et al. (2019). Briefly, extracts were filtered (0.45 μm, Thermo Fisher Scientific, San Jose, CA, USA) and analyzed using an Agilent 1,260 series reversed-phase high performance liquid chromatography (HPLC) system (Agilent 1,260, Santa Clara, CA, USA) coupled to a diode array detector. Separation was performed on a reversed-phase C18 column LiChrospher® 100, 250 × 4 mm with a 5 μm particle size and a 4 mm guard column of the same material at 25°C with a flow rate of 0.5 mL/min. Chromatographic conditions were previously reported in Martínez-Lüscher et al. (2019). Commercial standards of malvidin-3-*O*-glucoside and quercetin-3-*O*-glucoside (Sigma-Aldrich, St. Louis, MO, USA) were used for the quantification of anthocyanins and flavonols, respectively.

### AMF Colonization

Intraradical AMF colonization was measured after harvest of each season where root samples (mainly root hairs) from the three middle grapevines per treatment replicate were collected at a depth of 15 and 20 cm away from the vine trunk. Root samples were cleaned, cleared, and stained according to methods described in Koske and Gemma (1989). Fifty root segments per replicate (~1 cm each) were examined under the microscope to determine intraradical AMF colonization as previously described in Torres et al. (2016, 2021a). Briefly, the extension of mycorrhizal colonization was determined by estimating its product in width and length according to a scale range between 0 and 10, where 0 is the complete absence of fungal structures. Then, the incidence of mycorrhizal colonization was estimated by dividing the number of root segments with the presence of fungal structures and the total observed segments. The intensity of the colonization was calculated as the product between the extension and incidence, and the result was expressed as a percentage of colonization.

### WF Assessment

WF was calculated following the methods described in Zotou and Tsihrintzis (2017) with minor modifications. Briefly, WF was derived as the sum of the green, blue, and gray WFs and expressed in m^3^ of water consumed per tonne of fruit harvested. Green, blue, and gray components were given by following equations:

$$Eq.2.\;{\;}_{\text{green}}WF=\frac{\sum Pm}{Y}$$

where P_m_ is the monthly effective precipitation expressed in m^3^ · ha^−1^ after applying a conversion factor of 10 and Y is the yield of grapevines expressed in tonne·ha^−1^.

$$Eq.3.\;{\;}_{\text{blue}}WF=\frac{\sum WUm}{Y}$$

where WU_m_ is the total amount of irrigation water received by the grapevines monthly expressed in m^3−^. ha^−1^ and Y is the yield of grapevines expressed in tonne·ha^−1^

$$\begin{array}{l} Eq.4.\;{\;}_{\text{gray}}WF=\frac{\alpha AR}{\left(cmax-cnat\right)\;Y} \end{array}$$

where α is the percentage of fertilizer that leaches to the receiving aquatic system; AR is the amount of fertilizer applied to the grapevines expressed in kg · ha^−1^; c_max_ is the maximum acceptable concentration of fertilizer in the aquatic system (mg · L ^−1^); and c_nat_ is the natural concentration of the pollutant in the aquatic system (mg · L^−1^). P_m_ values were obtained from the CIMIS station placed in the vineyard. For gray component calculation, only nitrogen fertilization was considered, given the environmental issues derived from its use in agriculture (UC Davis, 2016). The percentage of nitrogen entering the water system of the area was assumed 10% according to Mekonnen and Hoekstra (2011). The maximum acceptable concentration of nitrogen (45 mg · L^−1^) was obtained from CDFA California Department of Food Agriculture (2020). According to Hoekstra et al. (2011), the natural concentration of pollutants was taken equal to zero, as proposed when data were missing.

### Statistical Analyses

Statistical analyses were conducted with R studio version 3.6.1 (RStudio: Integrated Development for R., Boston, MA, USA) for Windows. After normality assessment, data were submitted to an ANOVA to assess the statistical differences between the different irrigation treatments. Means ± SEs were calculated and when the F value was significant (*P* ≤ 0.05), a Tukey's “Honest Significant Difference” (HSD) *post hoc* test was executed by using “agricolae” 1.2–8 R package (de Mendiburu, 2016). Pearson correlation analyses were conducted with the same software.

## Results

### Weather Conditions

Mean day temperature and precipitation recorded by the CIMIS station are presented in Figure 1A. The measurements/records started in October of the previous year to account for the rain received during the dormant season. In the second year (2020) of the experiment, precipitation was five times less compared to the long term averages, with an exceptionally arid February. Mean day temperatures were similar between both years (14.5 and 14.9°C, respectively). However, the 2020 growing season had 17 more days with high temperatures (over 30°C). Therefore, irrigation in 2020 started 1 month earlier than in 2019 (Figures 1B,C). Cumulative water applied at the end of the season reflected the different water applications across treatments where 100% ET_c_ plots received 2,335 and 1,772 m^3^ · ha^−1^ more in 2019 and 2020 growing seasons, respectively, compared with 25% ET_c_ plots.

### Grapevine _i_WUE, WUE_c_, Must δ^13^C, Growth, and Yield Components Were Affected by Irrigation Treatments

**Figure 2:**
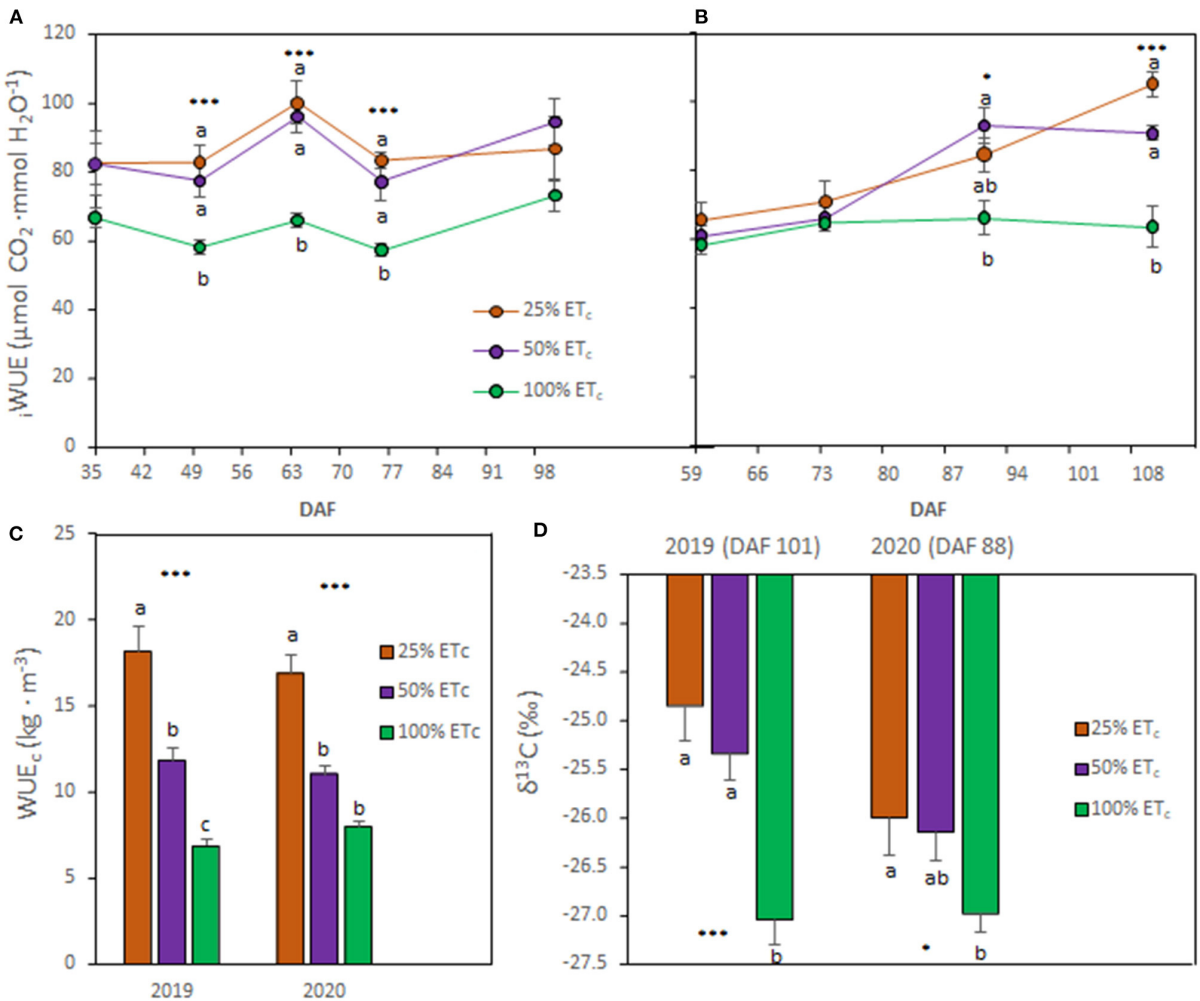
Intrinsic water use efficiency (iWUE, **A** and **B**), crop WUE (WUE_c_, **C**), and δ_13_C of berry must **(D)** of Cabernet Sauvignon grapevines (clone FPS08), subjected to different replacements of crop evapotranspiration (25, 50, and 100% ET_c_) and collected through 2018–2019 and 2019–2020 growing seasons in Oakville, CA. Values represent means ± SE (*n* = 6). At each time point, different letters indicate significant differences (*p* ≤ 0.05) between irrigation treatments according to one-way ANOVA followed by Tukey HSD test. *, and *** indicate significance at 5, and 0.1% probability levels, respectively. DAF, Days after flowering.

The 25 and 50% ET_c_ irrigation treatments increased the _i_WUE, starting around véraison (DAF 57 for 2019 and DAF 62 for 2020) until harvest (Figures 2A,B). At harvest, 25 and 50% ET_c_ improved the _i_WUE between 18.6 and 29.2% in 2019 and between 29.2 and 42.9% in 2020, respectively, compared with the 100% ET_c_ treatment. The WUE_c_ was higher with the 25 and 50% ET_c_ compared with 100% ET_c_ in 2019, whereas only 25% ET_c_ improved WUE_c_ in 2020 (Figure 2C). The berry must δ^13^C was greater in treatments subjected to higher water deficits (Figure 2D). Thus, berry must δ^13^C was higher in 25 and 50% ET_c_ treatments in 2019 while in 2020, only in 25% ET_c_ was significantly higher compared with 100% ET_c_ treatment.

Yield components and vegetative growth responded to irrigation treatments during both growing seasons (Table 1). Cluster mass decreased with 25% ET_c_, whereas 100% ET_c_ increased it. The number of clusters per vine ranged between 56 and 59 and between 53 and 55 in 2019 and 2020 growing seasons, respectively, and did not differ between treatments. Likewise, a significant enhancement of vegetative growth was measured by increases in LAI and total leaf biomass per area unit area with the 100% ET_c_. Yield to pruning mass ratio was only affected by treatments in the 2019 growing season where 25% ET_c_ decreased it compared with other treatments.

**Table 1:** Reproductive and vegetative growth of Cabernet Sauvignon grapevines (clone FPS08) subjected to different replacement of crop evapotranspiration (25, 50, and 100% ET_c_), collected in Oakville, CA, in 2018–2019 and 2019–2020 seasons.

	**Cluster mass**	**Leaf area index**	**Leaves**	**Yield:pruning mass**
	**(g)**	**(m^**2**^/m^**2**^)**	**(tonne/ha)**	**(kg/kg)**
2019				
Treatments				
25% ET_c_	121.7 ± 7.9 **c**	1.15 **b**	2.7 ± 0.1 **c**	4.1 ± 0.4 **b**
50% ET_c_	153.5 ± 9.1 **b**	1.25 **b**	4.2 ± 0.4 **b**	6.9 ± 0.7 **a**
100% ET_c_	176.4 ± 6.8 **a**	2.62 **a**	5.3 ± 0.2 **a**	6.9 ± 0.8 **a**
*ANOVA*	***	***	***	*
2020				
Treatments				
25% ET_c_	97.4 ± 6.8 **c**	1.49 **ab**	2.6 ± 0.3 **b**	7.3 ± 1.2
50% ET_c_	121.7 ± 5.5 **b**	0.91 **b**	3.0 ± 0.2 **b**	6.8 ± 0.9
100% ET_c_	170.0 ± 3.8 **a**	2.08 **a**	5.1 ± 0.5 **a**	5.6 ± 0.7
*ANOVA*	***	·	***	ns

Values represent means ± SE (n = 6) separated by Tukey HSD test (P ≤ 0.05). Different letters within column, indicate significant differences as affected by the different replacements of irrigation. ns, ^*^, and ^***^ indicate non-significance and significance at 10, 5, and 0.1% probability levels, respectively.

### Berry Composition Was Affected by Replacement of Different ET_c_ Fractions

**Figure 3:**
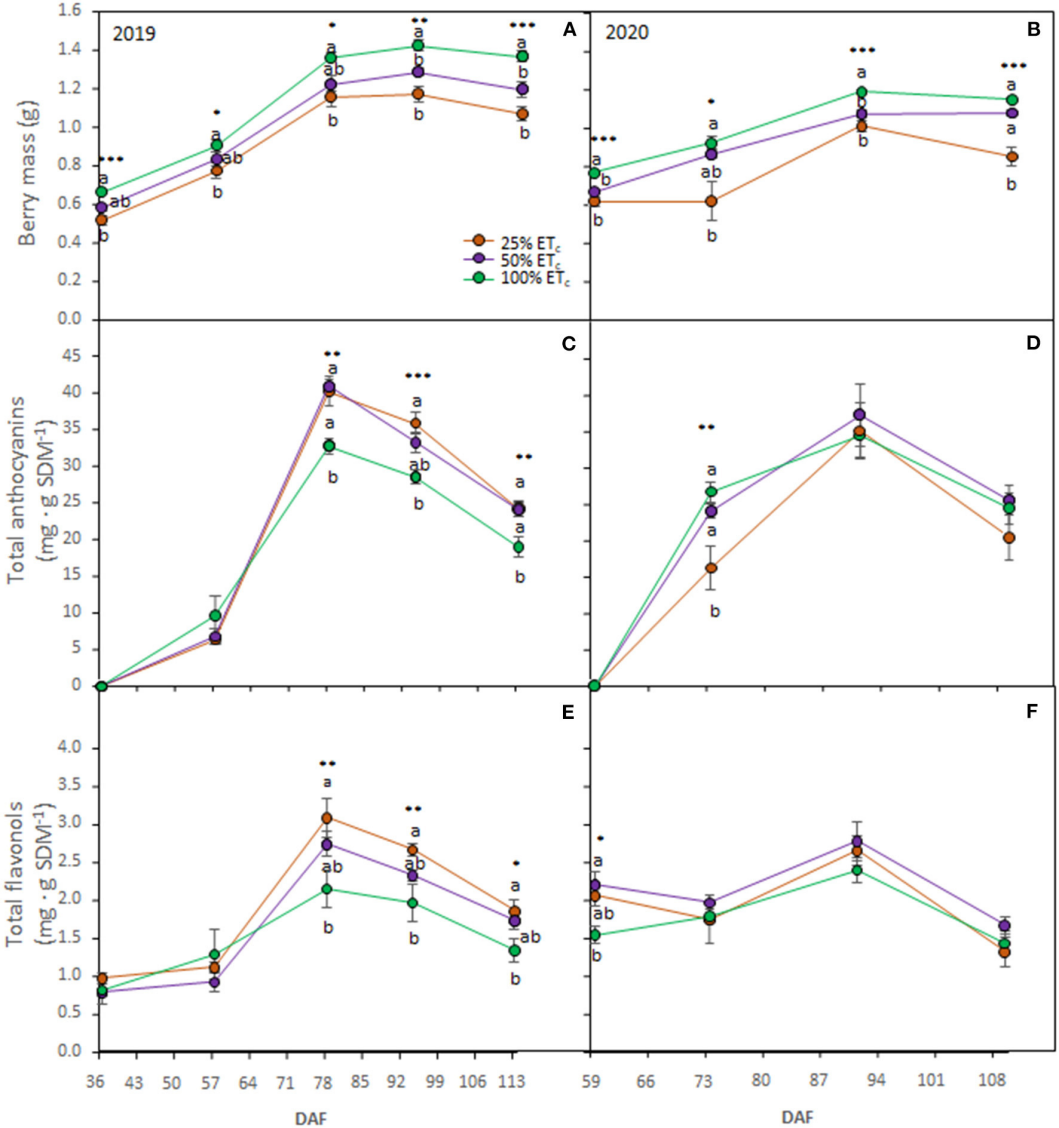
Berry mass (**A, B**), total anthocyanins (**C, D**), and total flavonols (**E, F**) in berry skin dry mass (SDM) of Cabernet Sauvignon grapevines (clone FPS08), subjected to different replacements of crop evapotranspiration (25, 50, and 100% ET_c_) and collected through 2018–2019 and 2019–2020 growing seasons in Oakville, CA. Values represent means ± SE (*n* = 6). At each time point, different letters indicate significant differences (*p* ≤ 0.05) between irrigation treatments according to one-way ANOVA followed by Tukey HSD test. *, **, and *** indicate significance at 5, 1, and 0.1% probability levels, respectively. DAF, Days after flowering.

Berry mass was affected by the applied water treatments during berry ripening in both seasons (Figures 3A,B) where it was the highest with the 100% ET_c_ treatment. We did not measure any treatment differences in TSS, pH, or TA in 2019 (Table 2). In the 2020 growing season, 100% ET_c_ treatment resulted in a lower TSS but higher must pH at harvest, compared with other treatments.

**Table 2:** Total soluble solids (TSS), titratable acidity (TA) and pH of Cabernet Sauvignon grapevines (clone FPS08) subjected to different replacement of crop evapotranspiration (25, 50, and 100% ET_c_), collected at harvest in Oakville, CA, in 2018–2019 and 2019–2020 seasons.

	**TSS (° Brix)**	**TA (g/L)**	**pH**
2019			
Treatments			
25% ET_c_	25.4 ± 0.3	7.12 ± 0.10	3.51 ± 0.02
50% ET_c_	24.7 ± 0.4	7.55 ± 0.33	3.45 ± 0.01
100% ET_c_	24.3 ± 0.4	7.37 ± 0.15	3.49 ± 0.02
*ANOVA*	ns	ns	ns
2020			
Treatments			
25% ET_c_	25.3 ± 0.4 **a**	9.13 ± 0.17	3.63 ± 0.01 **b**
50% ET_c_	25.0 ± 0.3 **a**	9.15 ± 0.23	3.65 ± 0.02 a**b**
100% ET_c_	23.8 ± 0.2 **b**	8.88 ± 0.21	3.69 ± 0.01 **a**
*ANOVA*	**	ns	**

Values represent means ± SE (n = 6) separated by Tukey HSD test (P ≤ 0.05). Different letters within column, indicate significant differences as affected by the different replacements of irrigation. ns and ^**^ indicate non-significance and significance at 1% probability level, respectively.

In 2019, the 100% ET_c_ decreased berry anthocyanin and flavonol contents from DAF 78 to harvest (DAF 114) (Figures 3C,E). In 2020, berry anthocyanin and flavonol contents were not affected by treatments except at prevéraison (DAF 59) where 100% ET_c_ decreased berry skin flavonol (Figure 3F). At véraison in 2020, the 25% ET_c_ had a significantly lower content of anthocyanins (Figure 3D) compared with other treatments.

Malvidin derivatives were the most abundant anthocyanins found in the Cabernet Sauvignon berry skins, ranging from 74 to 78% and 65 to 74% in 2019 and 2020 growing seasons, respectively (Table 3). The treatments strongly affected anthocyanin compounds in both seasons. In 2019, at harvest, 50 and 100% ET_c_ increased the proportion of peonidins and 25% ET_c_ had a significantly higher proportion of petunidin derivatives.

**Table 3:** Anthocyanin composition (%) of Cabernet Sauvignon grapevines (clone FPS08) subjected to different replacement of crop evapotranspiration (25, 50, and 100% ET_c_), collected at harvest in Oakville, CA, in 2018–2019 and 2019–2020 seasons.

	**Delphinidin (%)**	**Cyanidin (%)**	**Petunidin (%)**	**Peonidin (%)**	**Malvidin (%)**
2019					
Treatments					
25% ET_c_	9.82 ± 0.32	0.76 ± 0.05	8.63 ± 0.20 **a**	5.82 ± 0.22 **b**	74.97 ± 0.74
50% ET_c_	8.76 ± 0.17	0.68 ± 0.02	7.83 ± 0.08 **b**	5.87 ± 0.18 **b**	76.86 ± 0.23
100% ET_c_	8.78 ± 0.30	0.84 ± 0.01	7.55 ± 0.16 **b**	7.07 ± 0.28 **a**	75.75 ± 0.28
*ANOVA*	ns	ns	**	***	ns
2020					
Treatments					
25% ET_c_	12.7 ± 0.42	1.56 ± 0.06	9.86 ± 0.23	5.92 ± 0.16 **b**	70.36 ± 0.59
50% ET_c_	12.4 ± 0.72	1.63 ± 0.13	9.52 ± 0.30	6.40 ± 0.30 **ab**	70.06 ± 1.33
100% ET_c_	12.0 ± 0.60	1.61 ± 0.12	9.17 ± 0.23	6.82 ± 0.25 **a**	70.18 ± 1.13
*ANOVA*	ns	ns	ns	*	ns

Values represent means ± SE (n = 6) separated by Tukey HSD test (P ≤ 0.05). Different letters within column, indicate significant differences as affected by the different replacements of irrigation. ns, ^*^, ^**^, and ^***^ indicate nonsignificance and significance at 5%, 1%, and 0.1% probability levels, respectively.

Flavonol composition was only affected by irrigation treatments during the 2020 growing season at harvest (Table 4). Myricetin and quercetin derivatives were the main flavonols found in Cabernet Sauvignon berry skins and both accounted for about 75% of the total amount. The most restrictive applied water treatment increased proportion quercetins and kaempferols, while 100% ET_c_ increased myricetins and syringetins.

**Table 4:** Flavonol composition (%) of Cabernet Sauvignon grapevines (clone FPS08) subjected to different replacement of crop evapotranspiration (25, 50, and 100% ET_c_), collected at harvest in Oakville, CA, in 2018–2019 and 2019–2020 seasons.

	**Myricetins (%)**	**Quercetins (%)**	**Kaempferols (%)**	**Syringetins (%)**
2019				
Treatments				
25% ET_c_	37.46 ± 1.56	38.01 ± 1.90	6.10 ± 0.33	5.71 ± 0.49
50% ET_c_	38.16 ± 1.21	37.00 ± 1.38	5.66 ± 0.30	6.43 ± 0.36
100% ET_c_	38.04 ± 1.84	37.05 ± 1.82	5.23 ± 0.47	6.86 ± 0.40
*ANOVA*	ns	ns	ns	ns
2020				
Treatments				
25% ET_c_	30.32 ± 1.18 **b**	43.64 ± 1.06 **a**	8.37 ± 0.33 **a**	5.59 ± 0.23 **b**
50% ET_c_	33.29 ± 1.81 **ab**	40.54 ± 2.00 **ab**	7.45 ± 0.32 **a**	6.05 ± 0.26 **ab**
100% ET_c_	37.01 ± 1.45 **a**	38.56 ± 1.05 **b**	6.08 ± 0.39 **b**	6.49 ± 0.29 **a**
*ANOVA*	**	*	***	*

Values represent means ± SE (n = 6) separated by Tukey HSD test (P ≤ 0.05). Different letters within column, indicate significant differences as affected by the different replacements of irrigation. ns, ^*^, ^**^, and ^***^ indicate nonsignificance and significance at 5, 1, and 0.1% probability levels, respectively.

### Applied Water Amounts Influenced the Native Mycorrhizal Colonization of Grapevine Roots

**Figure 4:**
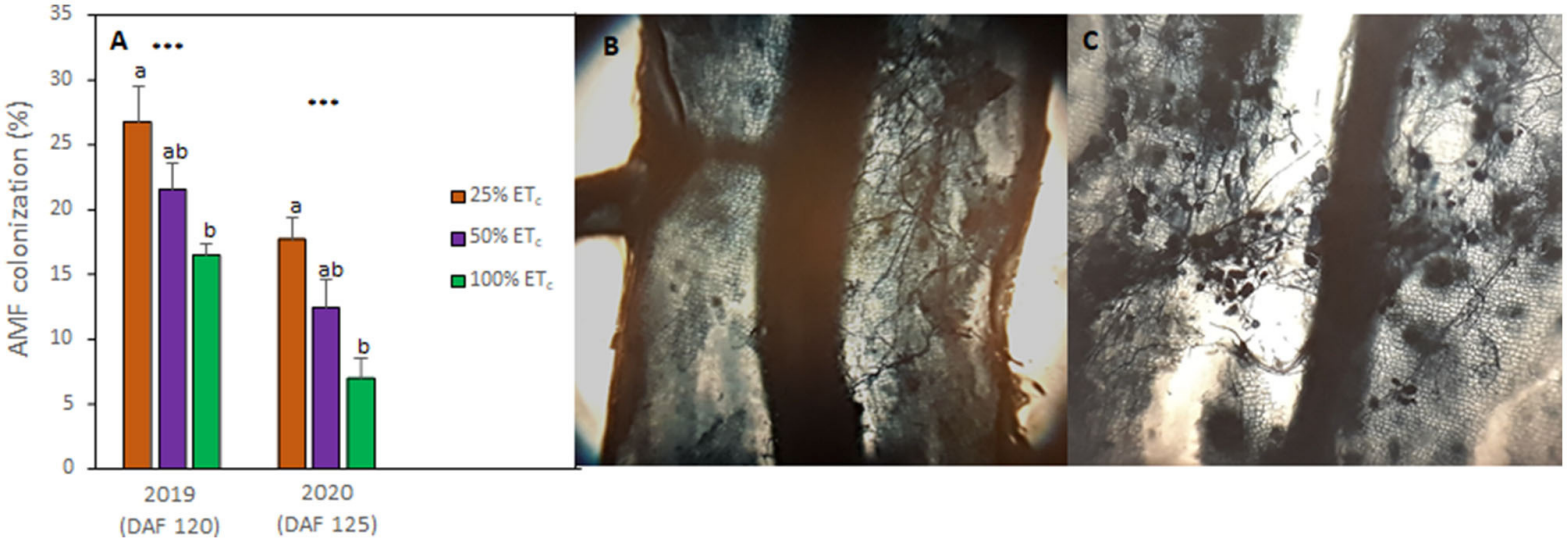
Arbuscular mycorrhizal fungi (AMF) colonization rates of Cabernet Sauvignon grapevines (clone FPS08) subjected to different replacement of crop evapotranspiration (25, 50, and 100% ET_c_), collected in Oakville, CA, in 2018–2019 and 2019–2020 growing seasons **(A)**. Bars represent means ± SE (*n* = 6). At each growing season, different letters indicate significant differences (*p* ≤ 0.05) between irrigation treatments according to one-way ANOVA followed by Tukey HSD test. *** indicate significance at 0.1% probability level. Microscopic image (×100) of fungal structures Cabernet Sauvignon roots present in half of the root fragment **(B)** and in the whole fragment **(C)**.

The analysis of native mycorrhizal colonization of grapevines indicated that 100% ET_c_ decreased the abundance of mycorrhizal structures compared with other treatments (Figure 4A). Thus, in 100% ET_c_, AMF colonization decreased by 10% in both growing seasons (Figure 4B) compared with the AMF colonization measured in 25% ET_c_ where the fungal structures were especially abundant (Figure 4C). The AMF colonization of 100% ET_c_ was 5% lower when compared with 50% ET_c_.

### Applied Water Amounts Affected the Total WF and Its Components

**Figure 5:**
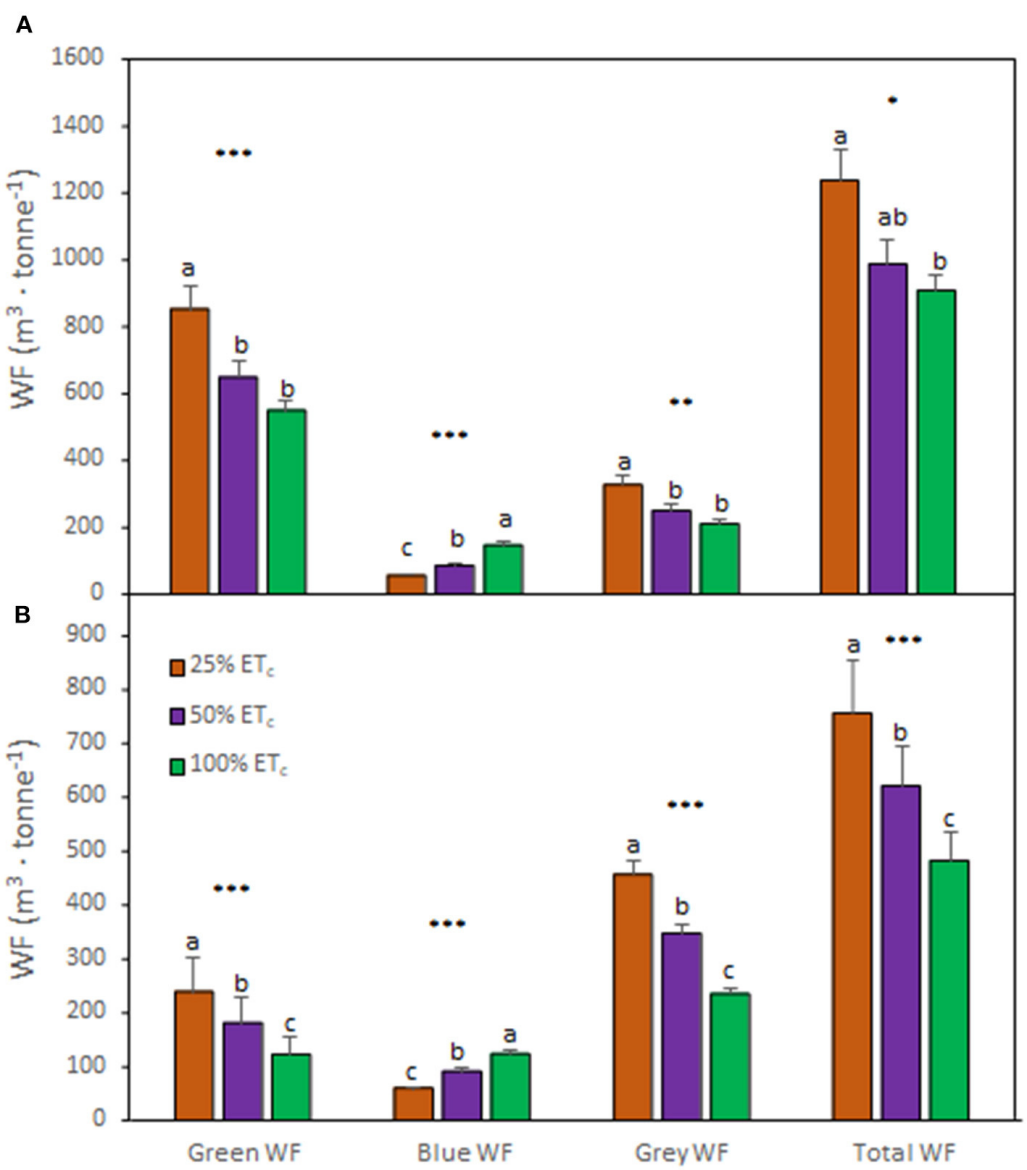
Water footprint (WF) components of Cabernet Sauvignon grapevines (clone FPS08) subjected to different replacement of crop evapotranspiration (25% ET_c_, 50% ET_c_, and 100% ET_c_), collected in Oakville, CA, in 2018–2019 **(A)** and 2019–2020 **(B)** growing seasons. Bars represent means ± SE (*n* = 6). At each WF component, different letters indicate significant differences (*p* ≤ 0.05) between irrigation treatments according to one-way ANOVA followed by Tukey HSD test. *, **, and *** indicate significance at 5, 1, and 0.1% probability levels, respectively.

Analysis of the effect of different applied water amounts on WF components indicated the same pattern regardless of the growing season (Figures 5A,B). The 25% ET_c_ increased the _green_WF and _gray_WF components and decreased the _blue_WF. Conversely, the 100% ET_c_ resulted in lower _green_WF and _gray_WF components and but higher _blue_WF. The _total_WF increased with the 25% ET_c_ treatment and decreased with the 100% ET_c_. This effect was more prominent during the second growing season (Figure 5B).

### Relationships Between Water Use and Grapevine Characteristics

**Figure 6:**
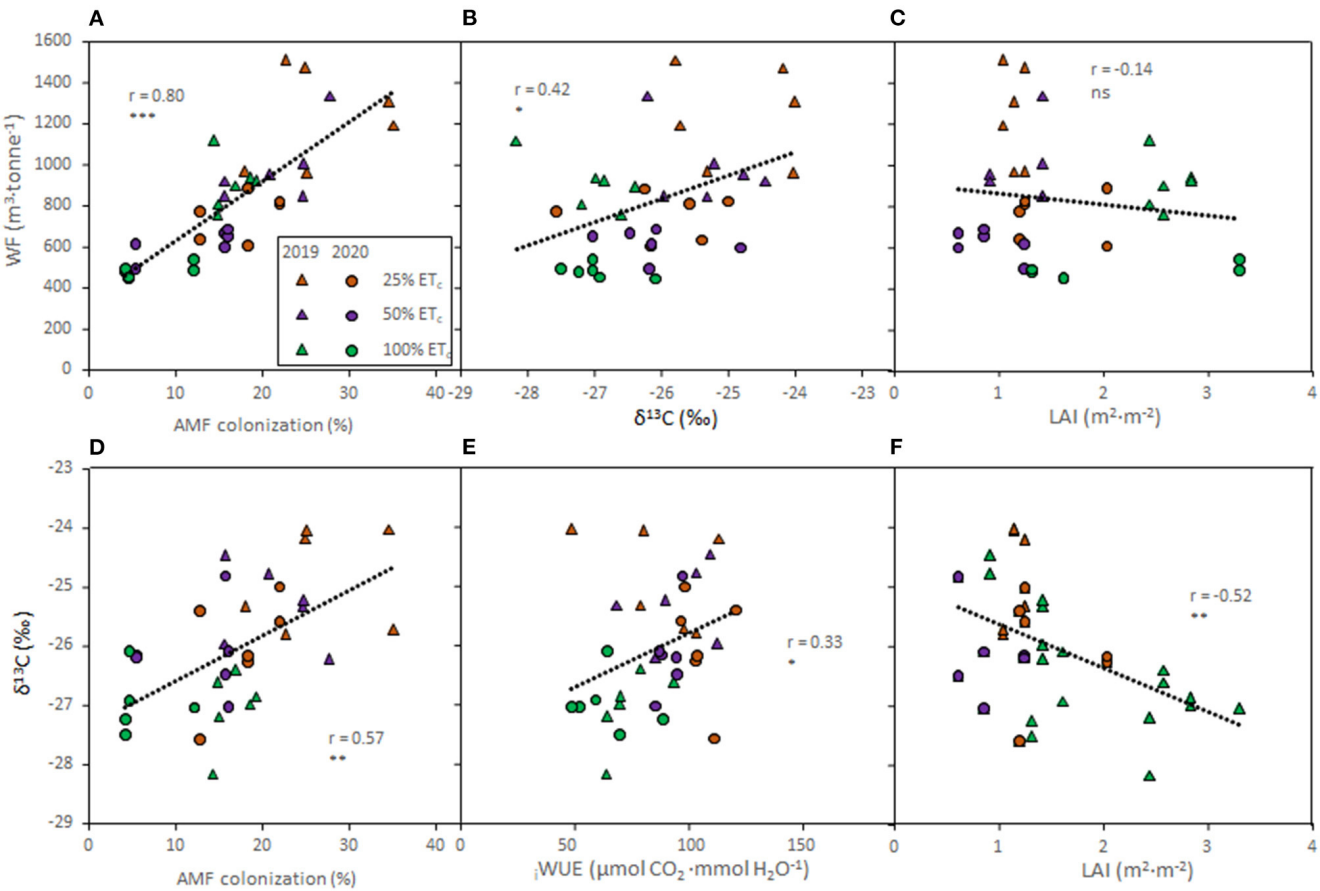
Relationships between total water footprint (WF) and arbuscular mycorrhizal fungi (AMF) colonization rates **(A)**; WF and δ_13_C of berry must **(B)**; and WF and leaf area index (LAI, **C**); δ_13_C of berry must and AMF **(D)**; δ_13_C and intrinsic water use efficiency (_i_WUE, **E**); and δ_13_C and LAI **(F)** of Cabernet Sauvignon grapevines (clone FPS08) subjected to different replacement of crop evapotranspiration (25, 50, and 100% ET_c_), collected in Oakville, CA, in 2018–2019 (triangles) and 2019–2020 (circles) growing seasons. For each combination, straight lines correspond to the linear regression lines fitted for the pooled data of all treatments in both seasons. ns, *, **, and *** indicate non-significance and significance at 5, 1, and 0.1% probability levels, respectively.

To determine relationships between water use and grapevine characteristics, correlation analyses were conducted on the pooled data of both seasons (Figure 6). The AMF colonization rates showed direct relationships with the _total_WF (Figure 6A, *r* = 0.80, *p* ≤ 0.0001) and with the δ^13^C of berry must (Figure 6D, *r* = 0.57, *p* ≤ 0.0001). The _total_WF was positively correlated with the δ^13^C berry must (Figure 6B, *r* = 0.42, *p* = 0.011). There was no discernable relation between LAI and the _total_WF (Figure 6C, *r* = −0.14, *p* = 0.432). However, an indirect relationship between LAI and the δ^13^C (Figure 6F, *r* = −0.52, *p* = 0.001) was evident. Finally, _i_WUE measured at harvest was positively correlated with the δ^13^C (Figure 6E, *r* = 0.33, *p* = 0.047).

## Discussions

### Improving WUE by Optimizing Applied Water Amounts and Their Effects on Berry Quality

This study evaluated the effect of applied water amounts based on the replacement of fractions of the ET_c_ for maintaining berry quality while minimizing yield losses due to the environmental impact (i.e., _total_WF and AMF colonization rates). Results covered two seasons that strongly differed in the precipitation supply. Compared with the average total amount of precipitation received by the area in the last decade (768 mm, CIMIS), 2019 growing season was a rainy period with 970.3 mm precipitation, while 2020 was a hyperarid growing season with only 234.2 mm of precipitation. In spite of the differences in total precipitation, the response of Cabernet Sauvignon grapevines to water deficits was consistent across both seasons and our results corroborated that deficit irrigation may mitigate the effects of water scarcity (Costa et al., 2016; Fraga et al., 2018; Torres et al., 2021b).

The results achieved in this study indicated that 25% and 50% ET_c_ treatments were effective in improving _i_WUE (*to ca*. 100 μmol CO_2_ ·mmol H_2_O^−1^) compared with previous studies reporting a compilation of data from Cabernet Sauvignon (_i_WUE ranged 62 and 64 μmol CO_2_ ·mmol H_2_O^−1^) and other cultivars (11.2–103 μmol CO_2_ ·mmol H_2_O^−1^) (Tomás et al., 2014; Bota et al., 2016). The _i_WUE decreased when the fractions of applied ET_c_ increased as previously reported by Keller et al. (2016) in a 3-year field experiment conducted on the same Cabernet Sauvignon clone as used in this study. Likewise, WUE_c_ calculated as the ratio between yield and water applied was also enhanced with the decreased water supply. The berry must δ^13^C enhanced under stronger water deficits conditions corroborating previous studies with different grapevine cultivars (Bchir et al., 2016; Spangenberg and Zufferey, 2018; Brillante et al., 2020; Yu et al., 2021). The _i_WUE and the berry must δ^13^C also indicated a linear relationship in accordance to previous research (Bchir et al., 2016). Previous work indicated that δ^13^C of grape must is a reliable indicator of plant water status and leaf gas exchange in vineyard systems, which in turn, are crucial for the identification of plant water status zones leading to better irrigation decisions and informed management (Brillante et al., 2020). The present study also provided evidence that δ^13^C is a convenient tool without intensive labor and time inputs for the assessment of environmental impacts (i.e., WF and/or AMF) of deficit irrigation strategies.

Increased applied water amounts led to greater canopy size and yields (Torres et al., 2021b). There was a strong negative relationship between the berry must δ^13^C and grapevine vegetative growth measured as LAI. Previous studies reported a linear relationship between the δ^13^C and the carbon assimilation rates (Bchir et al., 2016; Brillante et al., 2020) and consequently with vegetative growth estimated as pruning mass (Brillante et al., 2020; Yu et al., 2021). Yield achieved in this experiment ranged from 4.8 to 10.4 kg · vine^−1^ (Torres et al., 2021b) in accordance to a previous study conducted in a vineyard at a similar density (Munitz et al., 2020). Thus, 100% ET_c_ may double the yield compared with the 25% ET_c_ as previously reported by Keller et al. (2016). This suggested that the effect of applied water on yield components is consistent in spite of the climate difference, planting space, and grapevine age.

Under our experimental conditions, primary metabolites (i.e., sugars and organic acids) were affected by applied water amounts in the second season, where 100% ET_c_ accounted for lower TSS but higher pH. Increased water content in berries was associated with a lower concentration of sugars due to a dilution effect (Terry and Kurtural, 2011; Keller et al., 2016). Conversely, the lower pH in 25% ET_c_ grapevines was related to exacerbated organic acid degradation under high temperatures by water deficit (Martínez-Lüscher et al., 2017, 2020). Berry skin flavonol and anthocyanin contents decreased with the 100% ET_c_ in 2019 but not in 2020. Although several studies reported increases in berry flavonoid content under mild or moderate water deficit (Gambetta et al., 2020), field research conducted in California resulted in contradictory results when severe water deficits were combined with a long hang time (Brillante et al., 2018; Yu et al., 2020, 2021).

In general, 100% ET_c_ irrigation treatment reduced the proportion of petunidin derivatives and increased the proportion of peonidin derivatives leading to a decreased ratio between tri-hydroxylated and di-hydroxylated anthocyanins, which was suggested to be less chemically stable for winemaking purposes (Torres et al., 2020). Likewise, previous studies have reported an increase in the ratio between tri-hydroxylated and di-hydroxylated anthocyanins when grapevines were subjected to water deficits given the upregulation of the relevant anthocyanin biosynthetic genes (Castellarin et al., 2007; Martínez-Lüscher et al., 2014; Cook et al., 2015; Savoi et al., 2017). In addition, these forms were more persistent through hang time, making tri-hydroxylated flavonoids more abundant as maturity progressed (Brillante et al., 2017). Flavonol composition was modified by applied water amounts in 2020 growing, where proportions of myricetin and syringetin derivatives increased and quercetin and kaempferol derivatives decreased with 100% ET_c_. Given that quercetin and kaempferol are important antioxidants in red wines, this shift in the composition may impact the antioxidant properties of wine (Dabeek and Ventura-Marra, 2019; Torres et al., 2020). In previous work, it was reported that 100% ET_c_ irrigation increased the net carbon assimilation and improved the grapevine water status, leading to higher soluble sugar and starch contents in leaves with the highest yields, and vegetative biomasses (Torres et al., 2021b). However, the greatest leaf area to fruit ratios measured in this treatment showed a clear sign of disproportionate leaf biomass growth, which presumably impacted berry metabolism. Thus, both studies highlighted the importance of management of water deficits to ensure grape berry composition optimization, improving water use sustainability by rewarding quality over quantity in arid and semiarid regions (Medrano et al., 2015; Romero-Azorín and García-García, 2020).

### Reducing Environmental Costs Through Irrigation Management Optimization

Decreasing irrigation amounts increased AMF colonization in accordance with previous studies (Schreiner et al., 2007). The symbiotic relationship of AMF with grapevines provided several adaptive advantages, such as improved abiotic and biotic stress resistance, enhanced nutrient uptake, and grapevine growth (Trouvelot et al., 2015; Torres et al., 2018a). Previous research suggested that these effects might be related to the altered regulation of nutrient transport, cell wall-related, phenylpropanoid, and stilbene biosynthesis genes driven by AMF colonization (Bruisson et al., 2016; Balestrini et al., 2017). Additionally, it was recently reported that AMF may enhance the content of flavonoids in berries (Torres et al., 2019, 2021a), leading to improved berry composition and antioxidant properties in spite of the lack of effect on petiole nutrient contents (Torres et al., 2021a). However, vineyard management practices may affect the soil structure and the composition of the rhizosphere-living microbiota (Coller et al., 2019; Vink et al., 2021), as well as the microbiota associated with grapevine roots, which is mainly composed by *Rhizophagus* and *Glomus* genus (Schreiner, 2020), likely affecting the effectiveness of the symbiosis. The relationship between AMF and berry must δ^13^C suggested that productivity of high quality grapes could still be sustained in this region with less water input because the root system of the grapevines may perform more efficiently due to greater AMF colonization.

The _total_WF measured in this study ranged between 484.3 and 1237.7 m^3^ ·tonne ^−1^ across treatments and growing seasons, in accordance with previous studies assessing the WF of grapevine cultivation (Mekonnen and Hoekstra, 2011). This variation in the _total_WF was related to the amount applied (determining _blue_WF) and the differences in precipitation between the two seasons (determining _green_WF). Indeed, the previous research speculated that changes in temperature and precipitation may affect the proportional contribution of blue and green WF to the _total_WF (Zotou and Tsihrintzis, 2017). Previous studies reported that vineyards accounted for a higher WF compared with other crops such as olives, wheat, and other fruit trees (Zotou and Tsihrintzis, 2017; D'Ambrosio et al., 2020). Wine grape growers require appropriate irrigation schedules that reduce _blue_WF and increase _green_WF leading to a decreased _total_WF for increasing sustainability of vineyards. Under our experimental conditions, 25% ET_c_ strongly decreased the _blue_WF, however, this came with a dramatic increase in the _gray_WF component, which led to increased _total_WF. Conversely, 100% ET_c_ decreased _total_WF to lower values than those reported in Zotou and Tsihrintzis (2017), presumably because of the differences of standard yields recorded in Mesogeia area (Greece), where the authors conducted their research, and Napa Valley, CA, USA where this study was performed. A recent study reported that the current values of _blue_WF and _gray_WF are unsustainable (D'Ambrosio et al., 2020). The actual runoff of the surface water is not sufficient to satisfy the irrigation requirements and/or dilute the pollutant load associated with the diffuse and point sources to reduce it below the maximum acceptable concentration (D'Ambrosio et al., 2020). These results highlighted that the management of natural resources, specifically water management, is paramount for the sustainability of the wine industry under future constraints (Schultz, 2016; Romero-Azorín and García-García, 2020; Wilson et al., 2020). Thus, our data suggested that values ranging between 600 and 1000 m^3^·tonne ^−1^ of the _total_WF may ensure a high _i_WUE of grapevines (Bota et al., 2016), optimum LAI, and profitable yields, which maintained the balance between vegetative and reproductive growths (Torres et al., 2021b). Nevertheless, it is noteworthy to address that WF assessment also presents some limitations given that the water consumed by an irrigated crop is often a mix of residual soil moisture from previous precipitation and irrigation events (residual green and blue WFs) and that the reference ET (ET_o_) is strongly dependent on the local climate (Fereres et al., 2017).

## Conclusions

We aimed to evaluate the standard irrigation practices on grapevine WUE, berry flavonoid composition, vineyard WF, and AMF-grapevine symbiosis in two seasons with contrasting amounts of precipitation. Irrigation in grapevine vineyards mitigated the water scarcity when precipitation during the dormant season was not sufficient. This study provided field data supporting that despite the low rainfall recorded in one of the seasons, an increase in the amount of irrigation was not advised. Thus, irrigating grapevines with the replacement of the 50% ET_c_ was still adequate in spite of the warming trends. In this treatment, berry composition was improved with increased contents of TSS, anthocyanins, and flavonols, and a stable flavonoid profile without an economic decrease in yield. In addition, with 50% ET_c_, the mycorrhizal symbiosis was not compromised and water resources were not highly impacted. Altogether, this study provides fundamental knowledge for viticulturists to design an appropriate irrigation schedule under the future constraints.

## Data Availability Statement

The raw data supporting the conclusions of this article will be made available by the authors, without undue reservation.

## Author Contributions

SK conceived and designed the study and acquired the funding. NT, RY, JM-L, and EK executed the trial. NT and RY collected and curated the data. All authors contributed to the writing of the manuscript and approved the final version.

## Conflict of Interest

The authors declare that the research was conducted in the absence of any commercial or financial relationships that could be construed as a potential conflict of interest.

## Publisher's Note

All claims expressed in this article are solely those of the authors and do not necessarily represent those of their affiliated organizations, or those of the publisher, the editors and the reviewers. Any product that may be evaluated in this article, or claim that may be made by its manufacturer, is not guaranteed or endorsed by the publisher.
